# Life‐history predicts past and present population connectivity in two sympatric sea stars

**DOI:** 10.1002/ece3.2938

**Published:** 2017-04-23

**Authors:** Jonathan B. Puritz, Carson C. Keever, Jason A. Addison, Sergio S. Barbosa, Maria Byrne, Michael W. Hart, Richard K. Grosberg, Robert J. Toonen

**Affiliations:** ^1^ Marine Science CenterNortheastern UniversityNahantMAUSA; ^2^ Hawai'i Institute of Marine BiologySchool of Ocean and Earth Science and TechnologyUniversity of Hawai'i at MānoaKāne'oheHIUSA; ^3^ Department of BiologyKwantlen Polytechnic UniversitySurreyBCCanada; ^4^ Department of Biological SciencesSimon Fraser UniversityBurnabyBCCanada; ^5^ Department of BiologyUniversity of New BrunswickFrederictonNBCanada; ^6^ Schools of Medical and Biological SciencesUniversity of SydneySydneyNSWAustralia; ^7^ Crawford LabCentre for Evolutionary StudiesSimon Fraser UniversityBurnabyBCCanada; ^8^ Department of Evolution and EcologyCollege of Biological SciencesUniversity of California DavisDavisCAUSA

**Keywords:** gene flow, phylogeography, population genetics, population structure

## Abstract

Life‐history traits, especially the mode and duration of larval development, are expected to strongly influence the population connectivity and phylogeography of marine species. Comparative analysis of sympatric, closely related species with differing life histories provides the opportunity to specifically investigate these mechanisms of evolution but have been equivocal in this regard. Here, we sample two sympatric sea stars across the same geographic range in temperate waters of Australia. Using a combination of mitochondrial DNA sequences, nuclear DNA sequences, and microsatellite genotypes, we show that the benthic‐developing sea star, *Parvulastra exigua*, has lower levels of within‐ and among‐population genetic diversity, more inferred genetic clusters, and higher levels of hierarchical and pairwise population structure than *Meridiastra calcar*, a species with planktonic development. While both species have populations that have diverged since the middle of the second glacial period of the Pleistocene, most *P. exigua* populations have origins after the last glacial maxima (LGM), whereas most *M. calcar* populations diverged long before the LGM. Our results indicate that phylogenetic patterns of these two species are consistent with predicted dispersal abilities; the benthic‐developing *P. exigua* shows a pattern of extirpation during the LGM with subsequent recolonization, whereas the planktonic‐developing *M. calcar* shows a pattern of persistence and isolation during the LGM with subsequent post‐Pleistocene introgression.

## 1 Introduction

Life‐history traits, such as location of fertilization (i.e., broadcast spawning vs. direct sperm delivery), egg mass deposition (i.e., benthic vs. pelagic), and mode of larval development (i.e., feeding vs. nonfeeding), have long been thought to be the primary factors governing micro‐ and macro‐evolutionary processes and patterns in the sea (Jablonski, 1986, 2000; Palumbi, 1994; Strathmann, 1990, 1993). For benthic and sedentary species, larval dispersal often represents the only mechanism mediating population and genetic connectivity between disjunct habitats. It was commonly postulated that species with broadcast spawning and pelagic larval development will have higher connectivity between populations and less genetic structure across broad spatial scales than species with direct or benthic development (e.g., Hellberg, 1996; Todd 1998; Waples 1987).

However, several species display patterns of genetic structure that contrast with those predicted from life‐history traits (reviewed by Liggins, Treml, Possingham, & Riginos, 2016; Mercier, Sewell, & Hamel, 2013). For example, some species with planktonic larval development exhibit high levels of genetic structure, even at small spatial scales (Bird et al., 2007; Cowen, Lwiza, Sponaugle, & Paris, 2000; Karl & Avise, 1992; Mora & Sale, 2002; Puritz, Gold, & Portnoy, 2016; Rocha, Robertson, Roman, & Bowen, 2005; Taylor & Hellberg, 2003), and other species that lack planktonic larval development show complex patterns of genetic structure and population connectivity at regional scales (Johannesson, 1988; Marko, 2004; McGovern, Keever, Saski, Hart, & Marko, 2010; Sotka, Wares, Barth, Grosberg, & Palumbi, 2004; Todd et al. 1998). Additionally, recent applications of landscape (seascape)‐based analyses have shown that other factors such as habitat (Selkoe et al., 2010), ocean currents (White et al. 2010), and even proximity to coastal pollution (Puritz & Toonen, 2011) can significantly impact the population connectivity of marine species (reviewed in Selkoe et al., 2016). These studies highlight that sweeping generalizations about the role of pelagic larval duration are difficult to make and do not alone explain the observed variation among populations (Liggins et al., 2016; Riginos, Douglas, Jin, Shanahan, & Treml, 2011; Riginos & Liggins, 2013; Selkoe, Gaggiotti, Bowen, & Toonen, 2014; Weersing & Toonen, 2009). In short, genetic structure in the sea represents the outcome of a complex set of interactions among history, geography, fertilization, developmental mode, distribution of favorable habitat, transport processes, larval duration, and behavior, among other factors.

Studies comparing sympatric, closely related species that differ in the presence or absence of larval dispersal provide an especially informative window into the specific influence of life history on marine population connectivity by controlling for environmental factors that may also be impacting genetic structure (Dawson et al. 2014). Multiple studies have shown that the mode of larval dispersal can predict relative levels of genetic and phylogeographic structure in species comparisons (Dawson, Louie, Barlow, Jacobs, & Swift, 2002; Duffy, 1993; Haney, Dionne, Puritz, & Rand, 2008; Hellberg, 1996; Hoskin, 1997; Hunt, 1993; Lee & Boulding, 2009; Riginos & Victor, 2001; Sherman, Hunt, & Ayre, 2008). There are instances, however, where other ecological factors such as ocean currents, climate change, or ecological niche appear to override the influence of life history on genetic structure (Ayre, Minchinton, & Perrin, 2009; Iacchei et al., 2013; Kyle & Boulding, 2000; Marko, 2004; Selkoe et al., 2016).

Two intertidal sea stars found on the rocky shores of Australia have been the subject of multiple analyses of population genetic structure (Ayre et al., 2009; Hunt, 1993; Sherman et al., 2008). *Meridiastra calcar* and *Parvulastra exigua* both have extensive ranges that span most of southern and eastern Australia, with a large area of sympatric overlap where both species are abundant. While largely codistributed, these two species offer a stark contrast in modes of larval development (Byrne, 1992, 2006; Lawson‐Kerr & Anderson, 1978). *Meridiastra calcar*, the larger of the two species, is a gonochoric, broadcast spawning species with a lecithotrophic (nonfeeding) larval stage and a short larval duration of 1–2 weeks (Byrne, 1992, 2006). *Parvulastra exigua* lays externally fertilized, attached egg masses with benthic larval development (Byrne, 1995; Lawson‐Kerr & Anderson, 1978), and *P. exigua* has protandry‐like hermaphroditism with small individuals being predominantly male and large individuals being predominantly female with a few simultaneous hermaphrodites present (Barbosa, Klanten, Jones, & Byrne, 2012; Byrne, 1991). Some individuals self‐fertilize and their embryos develop to the juvenile stage (Barbosa et al., 2012).

Given these differences in reproductive and developmental modes, all else being equal, *P. exigua* should have higher levels of population differentiation across smaller spatial scales and lower levels of within‐population genetic diversity than *M. calcar*, especially across parts of their respective ranges where they co‐occur. As expected, a study using allozymes showed that *P. exigua* has lower levels of among‐ and within‐population genetic diversity and higher levels of population subdivision than *M. calcar* across a spatial scale of <200 km (Hunt, 1993). A more extensive allozyme‐based regional study spanning New South Wales to Tasmania reported a similar pattern (Sherman et al., 2008), as did Barbosa, Klanten, Puritz, Toonen, and Byrne (2013) using the putative mitochondrial control region between rRNAs (12S–16S), with the latter study showing population subdivision at a very small scale (50 m). In contrast, a recent study using mtDNA COI showed that *M. calca*r exhibited significant phylogeographic structure across a major biogeographic barrier (Bass Strait) in southeastern Australia, whereas *P. exigua* unexpectedly did not (Ayre et al., 2009). On the surface, these studies present differing patterns of population structure; however, the different marker types may actually be revealing different aspects of population structure. Studies using different marker types have helped to clarify different aspects of population structure in a variety of systems, and highlight the need for a comprehensive study combining the results from multiple marker types for *P. exigua* and *M. calcar*. Multiple markers allow for more robust analyses of population structure and can be effectively used to infer critical features such as directional migration rates, effective population size, and population divergence time (Hart & Marko, 2010; Lowe & Allendorf, 2010; Marko & Hart, 2011).

Here, we present results from a multilocus, comparative phylogeographic analysis of *M. calcar* and *P. exigua* to reconstruct their history. We show that these two species differ profoundly in the relative times of population divergence across major biogeographic barriers and also in the magnitude of migration between biogeographic regions. Based on the combined data from mtDNA sequences, microsatellite loci, and nuclear intron sequences, we test how well each of these species conform to expectations based on life‐history mode. We conclude that inferred gene flow and genetic diversity of these two species conform better to previous work using allozymes than to conclusions based solely on mitochondrial COI data.

## 2 Materials and Methods

### 2.1 Study area

**Figure 1:**
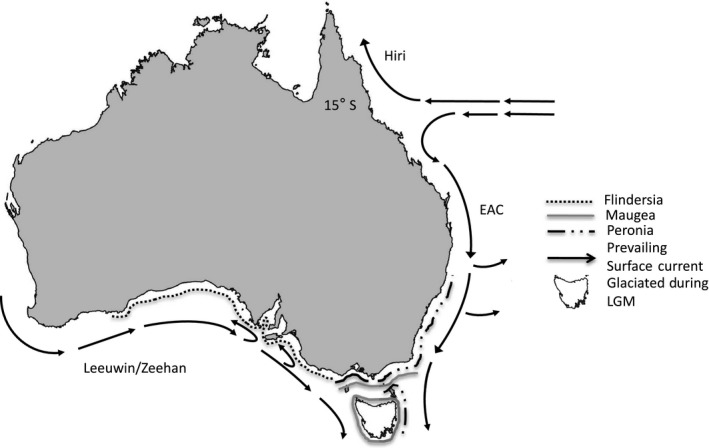
Map of Australia with major currents and biogeographic provinces. Note that Tasmania was only partially glaciated during the Pleistocene with glaciers restricted to high elevation plateaus

**Figure 2:**
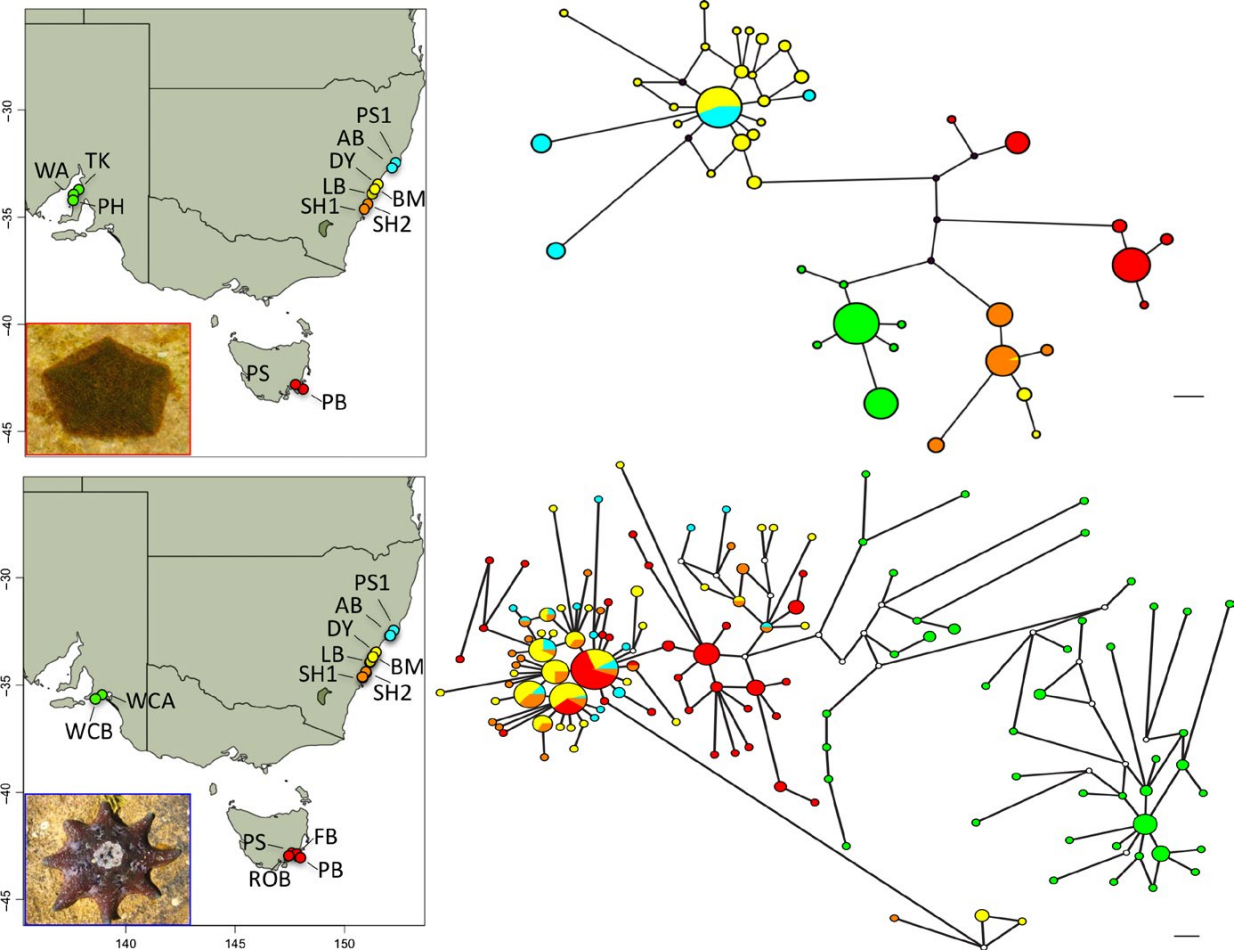
Map of sampling locations and mtDNA haplotype networks. *Parvulastra exigua* on top and *Meridiastra calcar* on the bottom. Networks are colored by region identically to the sampling sites on the maps, and branch lengths are proportional to genetic distance among haplotypes. Scale bars on the bottom right of each network image represent one mutational change. Abbreviations and regions are defined in Table 1

The southeastern portion of coastal Australia is divided into three biogeographic regions: Flindersian, Maugean and Peronian (Figure 1; Bennet & Pope, 1953). These regions are maintained by the contemporary interactions of the Eastern Australia Current (EAC) moving warm surface waters from north to south along the New South Wales (NSW) coastline and the Leeuwin Current transporting waters from west to east along portions of southern Australia (SA), as well as the Pleistocene land bridge between Tasmania (TAS) and mainland Australia (Reviewed in Ayre et al., 2009; Dawson, 2005; Sherman et al., 2008; Waters, 2008). We collected samples from 12 populations of *P. exigua* and 13 populations of *M. calcar* from five different geographical regions (Table 1; Figure 2, northern New South Wales, central New South Wales, southern New South Wales, Tasmania, and southern Australia). This sampling regime spans all three identified biogeographic regions and includes most of the sympatric range of both species (Figure 1).

**Table 1:** Locations, site codes, sample numbers, and molecular diversity of sampled populations. Diversity indices are *n*, number of samples; *H*_E_, expected heterozygosity; *H*_O_, observed heterozygosity; *N*_A_, number of alleles; *HD*, haplotype diversity, and π, average number of pairwise differences. For microsatellites, diversity measures are jackknifed across loci

Region	Sample location	Latitude	Longitude	Microsatellites				mtDNA			nDNA			
				*n*	*H*_E_	*H*_O_	*N*_A_	*n*	*HD*	π	*n*	*H*_E_	*H*_O_	*N*_A_
*Parvulastra exigua*														
NNSW	Port Stephens (PS1)	32.74815°S	152.17371°E	45	0.00	0.00	1.0	20	0.44	5.3	15	0.08	0.09	1.4
	Alison Beach (AB)	32.79036°S	152.11235°E					7	0.48	5.2	8	0.09	0.00	1.2
CNSW	Deewhy (DY)	33.77948°S	151.29489°E	40	0.15	0.10	2.0	19	0.91	4.2	20	0.14	0.12	1.6
	Balmoral Beach (BM)	33.82320°S	151.25296°E	44	0.11	0.11	1.8	18	0.11	0.1	19	0.19	0.12	1.6
	Little Bay (LB)	33.97977°S	151.25165°E	48	0.07	0.06	1.8	19	0.94	5.5	20	0.19	0.16	1.6
SNSW	Shell Harbour 2 (SH2)	34.54635°S	150.87946°E	32	0.17	0.13	2.6	15	0.00	0.0	19	0.19	0.17	1.8
	Shell Harbour 1 (SH1)	34.58026°S	150.87523°E	45	0.16	0.12	2.4	19	0.68	1.4	20	0.06	0.07	1.6
TAS	Pirates Bay (PB)	43.00768°S	147.93703°E	28	0.11	0.12	1.6	19	0.44	3.6	19	0.16	0.15	1.6
	Primrose Sands (PS)	42.88786°S	147.65386°E	41	0.20	0.15	2.0	20	0.72	6.5	16	0.11	0.07	1.8
SA	Port Hughes (PH)	34.07558°S	137.54586°E	20	0.02	0.02	1.2	19	0.00	0.0	20	0.06	0.07	1.2
	Wallaroo (WA)	33.92780°S	137.62055°E					20	0.44	0.6	19	0.16	0.15	1.6
	Tickera (TK)	33.78382°S	137.70814°E	30	0.05	0.04	1.2	18	0.00	0.0	19	0.00	0.00	1.0
*Meridiastra calcar*														
NNSW	Port Stephens (PS1)	32.74815°S	152.17371°E	48	0.49	0.41	7.4	21	0.92	7.1	20	0.95	0.54	24.6
	Alison Beach (AB)	32.79036°S	152.11235°E	40	0.51	0.40	6.1	20	0.96	3.0	20	0.97	0.55	23.4
CNSW	Deewhy (DY)	33.77948°S	151.29489°E	39	0.49	0.40	6.1	24	0.95	8.3	20	0.98	0.54	23.8
	Balmoral Beach (BM)	33.82320°S	151.25296°E	32	0.43	0.33	5.7	19	0.92	3.0	20	0.96	0.65	24.0
	Little Bay (LB)	33.97977°S	151.25165°E	40	0.42	0.40	5.4	23	0.94	4.8	20	0.98	0.66	25.0
SNSW	Shell Harbour 2 (SH2)	34.54635°S	150.87946°E	50	0.45	0.34	6.6	18	0.97	6.6	20	0.94	0.54	23.0
	Shell Harbour 1 (SH1)	34.58026°S	150.87523°E					19	0.95	5.2	19	0.95	0.65	22.6
TAS	Pirates Bay (PB)	43.00768°S	147.93703°E	40	0.58	0.44	7.4	20	0.88	3.6	20	0.95	0.53	22.8
	Fortescue Bay (FB)	43.14255°S	147.96403°E	30	0.56	0.48	5.3	20	0.83	3.1	19	0.95	0.61	19.8
	Primrose Sands (PS)	42.88786°S	147.65386°E	32	0.50	0.44	6.4	17	0.93	7.3	20	0.96	0.57	23.2
	Roaches Beach (ROB)	42.88936°S	147.50195°E					21	0.91	5.7	20	0.94	0.63	22.4
SA	Middletown (WCA)	35.51478°S	138.70731°E	30	0.42	0.33	5.6	30	0.94	12.5	20	0.96	0.55	22.2
	Waitpinga (WCB)	35.60263°S	138.58171°E	30	0.42	0.35	5.0	30	0.99	15.0	20	0.98	0.56	20.4

### 2.2 Mitochondrial DNA amplification

Following the protocols in Keever et al. (2009), we PCR‐amplified and sequenced two portions of the mitochondrial (mtDNA) genome. The tRNA/COI fragment was 703 bp for *P. exigua* and 714 bp for *M. calcar*. The putative mitochondrial control region was amplified following the protocols in Barbosa et al. (2013) and was 603 and 644 bp in each species, respectively. PCR conditions were 95**°**C for 3 min followed by 35 cycles of 94**°**C for 30 s, 57**°**C for 30 s, and 72**°**C for 60 s, and then a final 72**°**C extension for 5 min. For all analyses, loci were concatenated to make a single mtDNA locus of 1,350 bp (*M. calcar*) and 1,306 bp (*P. exigua*) respectively.

### 2.3 Microsatellite genotyping

We genotyped samples of *P. exigua* at five microsatellite loci and samples of *M. calcar* at seven microsatellite loci, following the protocols in Keever, Sunday, Wood, Byrne, and Hart (2008), Keever et al. (2009). Because of the work required to genotype every sample, we reduced the number of sampled localities used for genotyping by removing some regional replicate localities while increasing within‐locality sample sizes (Table 1; Fig. 2).

### 2.4 Next‐generation sequencing

We sequenced approximately 20 individuals from 12 localities of *P. exigua* and 13 of *M. calcar* at each of five nuclear intron (nDNA) loci as described in Puritz, Addison, and Toonen (2012) and Puritz and Toonen (2013). In short, for each individual, raw sequencing reads were demultiplexed, trimmed according to base quality, and mapped to reference sequences for each locus in the program geneious (version 7.1.7; Kearse et al. 2012). Due to large amounts of INDEL variation and INDEL‐like errors of 454 sequencing, many alignments were realigned by eye. If heterozygous base pairs were found in the contig alignment, the alignment was then sorted by that base pair. The two most common haplotypes were then selected as the two allelic states for that locus.

### 2.5 Molecular diversity indices

We calculated mitochondrial gene diversity and number of average pairwise differences in arlequin 3.5 (Excoffier & Lischer, 2010). We used genodive 2.0b21 (Meirmans & Van Tienderen, 2004) to calculate observed heterozygosity, expected heterozygosity, and average allelic diversity.

### 2.6 Phylogenetic analysis

We used the median‐joining and MP algorithms of NETWORK 4.6 (Bandelt, Forster, & Röhl, 1999; Polzin & Daneshmand, 2003) to construct phylogenetic networks.

### 2.7 Genetic structure analyses

modeltest 3.7 (Posada and Crandal 1998) selected the Tamura–Nei model as the best fit for genetic distance among mtDNA haplotypes. We then calculated all AMOVA and pairwise measures of population structure for mtDNA in arlequin 3.5 using this model, and 50,000 permutations for significance testing. We used genodive to calculate all AMOVA and pairwise measures of population structure for microsatellites using the standardized $F_{\mathrm{ST}}^{\prime }$ (AMOVA) and $G_{\mathrm{ST}}^{\prime \prime }$ (pairwise) statistics with 10,000 permutations for significance testing (Meirmans, 2006; Meirmans & Van Tienderen, 2004).

### 2.8 Bayesian clustering

We used TESS 2.3 (Chen, Durand, Forbes, & François, 2007; Durand, Jay, Gaggiotti, & François, 2009) to estimate genetic clusters from microsatellite genotypes integrated with spatial sampling information. For *P. exigua*, clusters were estimated without admixture based on the results of previous studies (Hunt, 1993; Sherman et al., 2008) using nuclear genes indicating very low levels of connectivity at both regional and fine spatial scales. 99,999 steps of burn‐in and 100,000 sweeps were used for 2–8 groups (the maximum range allowed by the program), with three replicates per group. Replicate runs in which *K* = 6 had the lowest deviance information criterion (DIC) score and results displayed are from the single lowest DIC run from the *K* = 6 replicates, and other replicates were consistent with the presented results. For *M. calcar*, clusters were estimated with the BYM admixture model (described in Durand et al., 2009), with spatial interaction optimization using a 999,999 step burn‐in and 1,000,000 sweeps for 2–6 groups with three replicates per group. Although runs with *K* = 5 had the lowest DIC score, we selected the *K* = 4 run with the lowest DIC as the best model. The ΔDIC from 4 to 5 was only 150.6 for the top runs, and the fifth genetic grouping was only present in a few individuals at very low percentages across a few populations.

We also estimated genetic clusters with structure 2.3.3 (Pritchard, Stephens, & Donnelly, 2000), using source population as a prior (Hubisz, Falush, Stephens, & Pritchard, 2009). For *P. exigua*, clusters, we used the “nonadmixture” mode (see above), with correlated allele frequencies, 100,000 burn‐in steps, 100,000 sampling steps from 1 to 7 groups, and three replicate runs per group. structure harvester 0.6.8 (Earl, 2012) was used to implement the Δ*K* method (Evanno, Regnaut, & Goudet, 2005), which identified *K* = 6 clusters as the best fit to the data. For *M. calcar*, clusters were estimated with admixture using correlated allele frequencies, 500,000 burn‐in steps, and 500,000 sampling steps from 1 to 5 groups, using three replicate runs per group. Δ*K* was highest for *K* = 2, but the data suggested that the relatively high Δ*K* for this small number of groups was artificially inflated by the relatively low negative log‐likelihood values from runs with *K* = 1. The likelihood score approached an asymptote at *K* = 3 (Figure S1), and removing *K* = 1 from the analysis resulted in *K* = 3 producing the highest the Δ*K* value. For these reasons, we chose *K* = 3 as the best‐fit model for *M. calcar*. Results from both species were summarized across replicates using clumpp (Jakobsson & Rosenberg, 2007) and visually displayed using distruct, grouped by population instead of individual (Rosenberg, 2003).

### 2.9 Coalescent analysis

We included all six sequence loci (the exception is for locus TBP in *P. exigua* which was fixed across all populations) for coalescent analyses in IMa2p (Hey, 2010; Sethuraman & Hey, 2016). We used the mtDNA phylogenetic networks to estimate population trees. IMa2 used a multipopulation model to calculate effective population size, migration rates, and divergence times between the sampled populations, and we conducted separate analyses for each species.

For each species, data sets were reduced to minimize the coalescent parameter space of six unlinked sequence loci (to increase likelihood of convergence in coalescent analyses). For *P. exigua*, only samples that had no missing data across the five nDNA loci and mtDNA locus were included and samples within northern NSW (AB, PS1; labeled as AB), within central NSW (DY, LB, BM; labeled as NSW), within southern NSW (SH1, SH2; labeled as SH), and within WA (WA, TK; labeled as SA) were combined and randomly subsampled into representative populations. For *M. calcar*, only samples that had no missing data across the five nDNA loci and mtDNA locus were included; samples from within northern and central NSW (AB, PS1, DY, LB, BM; labeled as NSW), within southern NSW (SH1, SH2; labeled as SH), within eastern TAS (FB, PB; labeled as FBPB), within western TAS (PS, ROB; labeled as PSROB), and within WA (WCB, WCA; labeled as SA) were combined and randomly subsampled down to 10 individuals with equal representation among localities.

20–30 preliminary runs for each species were conducted adjusting priors between runs to obtain the priors used in final analysis. MCMCMC analyses were run for burn‐in until flat trend lines appeared in all estimated parameters, and afterward 300,000 genealogies per locus were saved with 10 steps between genealogy saves. Joint peak locations and posterior probabilities were estimated with a single “L‐mode” run for each species. Initial runs of *P. exigua* returned abnormally large estimates of the mutation rate scalar (~24) for the mtDNA locus, likely because of the large difference in the number of haplotypes for the nDNA loci compared to mtDNA locus. For the final run, a geometric mean of all mutation rate scalars was used instead. This scalar more closely matched the scalar estimated for *M. calcar* and produced results similar to using the mtDNA locus alone for estimates.

### 2.10 Choosing a mutation rate for coalescent analysis

We used an estimated mutation rate for the concatenated mtDNA sequences of 9.9256 × 10^−6^ mutations per gene per year for *P. exigua* and 1.026 × 10^−5^ per gene per year for *M. calcar*. These rates are based on the smallest average pairwise sequence divergence between geminate pairs of Neotropical sea urchin species (another echinoderm) separated by the Isthmus of Panama (4.56% COI divergence between *Diadema mexicanum* and *Diadema antillarum*; Lessios et al. 2001), assuming 3 million years of isolation, which gives a rate of 7.60 × 0^−9^ mutations per site per year. We use an average COI rate across the entire locus, although we recognize that the concatenated mtDNA sequences in our study include small portions of slower evolving tRNA sequences and large portions of the faster evolving control region of the mitochondrion (Avise, 2004). A comparative study (Hickerson et al. 2006) concluded that geminate sea urchin species pairs diverged in two separate events and that geminate *Diadema* species diverged about 1 million years later than geminate pairs of other genera. In that case, our mutation rate estimate based on the least divergent geminate species pair ought to be relatively conservative. Likewise, if mutation rate estimates prove to be time dependent and higher for rates estimated from more recent calibration events (as argued by Crandall et al. 2012; Ho et al. 2011), a faster rate based on 4.56% divergence during a shorter period of isolation between *Diadema* species might be appropriate. This higher rate would tend to reduce the divergence times we estimate between *Meridiastra* or *Parvulastra* populations, but would not bias our comparison of the two species relative to each other.

## 3 Results

### 3.1 Genetic diversity

**Figure 3:**
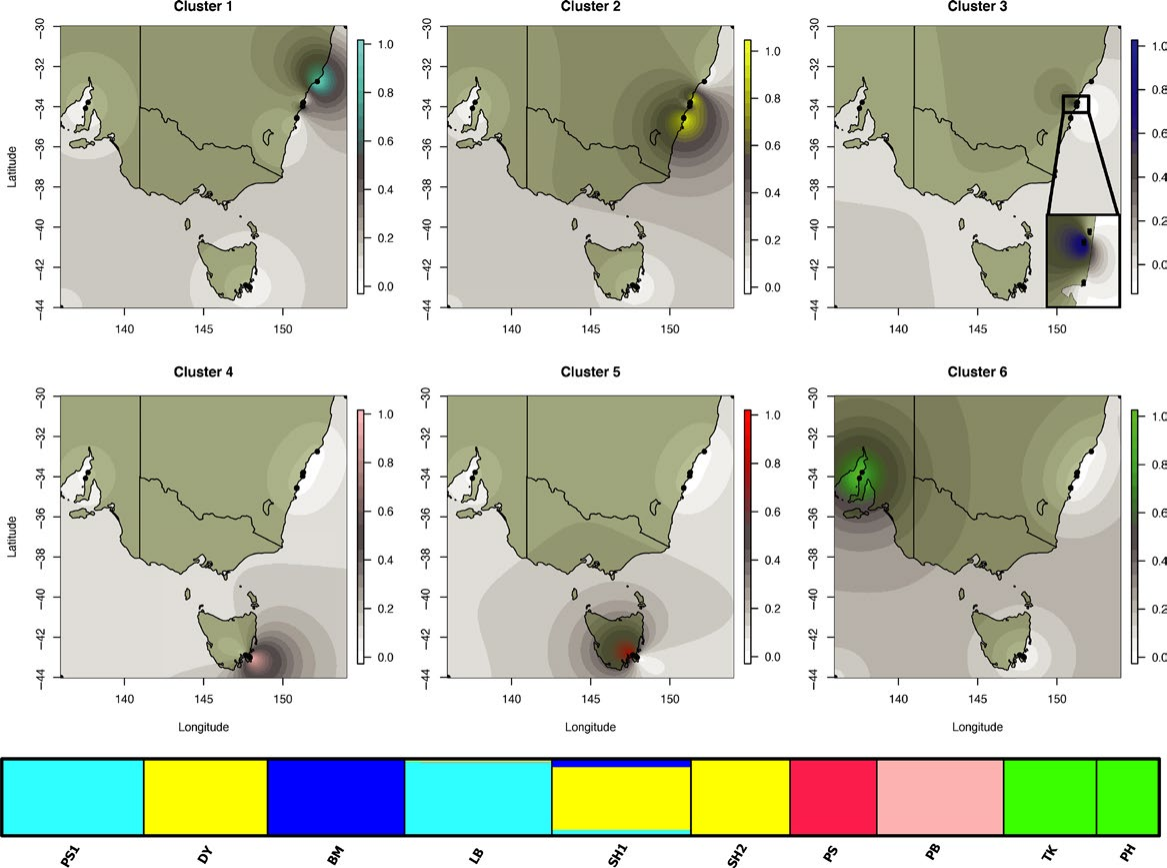
Bayesian clustering results for *Parvulastra exigua*. Top six panels are spatial interpolations of the six genetic clusters detected by TESS. The bottom panel are the *K* = 6 results from STRUCTURE plotted at the population level

Across all sampled loci, *M. calcar* has substantially higher levels of genetic diversity than *P. exigua*, and higher levels of within‐ and among‐population heterozygosity, allelic and haplotype diversity, and average pairwise distances between alleles (Figure 3; Table 1). Additionally, genetic diversity of *M. calcar* was similar to other asterinid species that have longer‐lived planktonic larvae (Keever et al., 2009; Puritz & Toonen, 2011). In contrast, most genetic diversity in *P. exigua* was distributed among populations with little diversity within single populations. Many populations had only a few mtDNA haplotypes, with some fixed for single haplotypes, and only a few different microsatellite genotypes (e.g., one sample of 45 individuals from PS1 all shared the same multilocus genotype).

### 3.2 Haplotype networks

The haplotype networks for both species revealed distinct regional partitioning of mtDNA genetic variation, but the details differ between the two.

For *P. exigua*, few haplotypes were shared between regions, and there was complete lineage sorting among SA, NSW, and WA. Alleles were shared between northern and central NSW as well as central NSW and southern NSW, but no haplotypes were present in all NSW populations (Figure 2).

In contrast, several haplotypes from *M. calcar* occurred in all three NSW regions and in the TAS samples, whereas WA was completely distinct from all other regions. The haplotypes also showed a partitioning of network characteristics. For example, NSW and TAS showed more starburst patterns, with most haplotypic diversity contributed by singletons that differed by only one mutation from a far more common central haplotype (Figure 2). In contrast, many haplotypes within SA occurred at low frequencies throughout the region and tended to be several mutations different from each other.

### 3.3 Bayesian population clustering

Both clustering methods produced similar results for *P. exigua* (Figure 3), showing six distinct genetic clusters. These clusters corresponded to the geographic partitioning shown in the mtDNA networks, but also revealed fine‐scale, localized structure. The three central NSW populations each fell into distinct groups. DY, the northernmost population of the three, clustered with the southern NSW populations (SH1 and SH2). BM fell in its own genetic cluster, and LB grouped with the northern NSW population, PS1.

**Figure 4:**
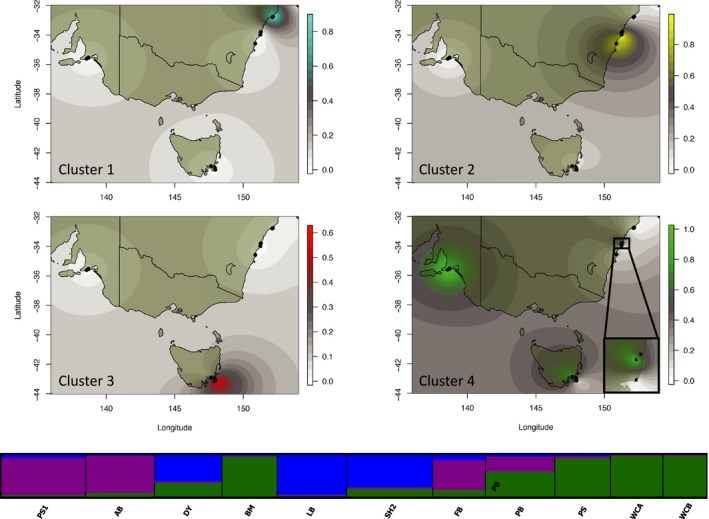
Bayesian clustering results for *Meridiastra calcar*. Top six panels are spatial interpolations of the four genetic clusters detected by TESS. The bottom panel are the *K* = 3 results from STRUCTURE plotted at the population level

In *M. calcar*, adding spatial information to genetic clustering increased the number of inferred genetic clusters from three to four (Figure 4). Interestingly, even with adding spatial priors, a population from central NSW (BM) and the western populations from TAS were grouped with the SA populations suggesting some degree of gene flow across a large stretch of the Southern Ocean and the Tasman Sea.

### 3.4 Pairwise and hierarchical genetic structure

**Figure 5:**
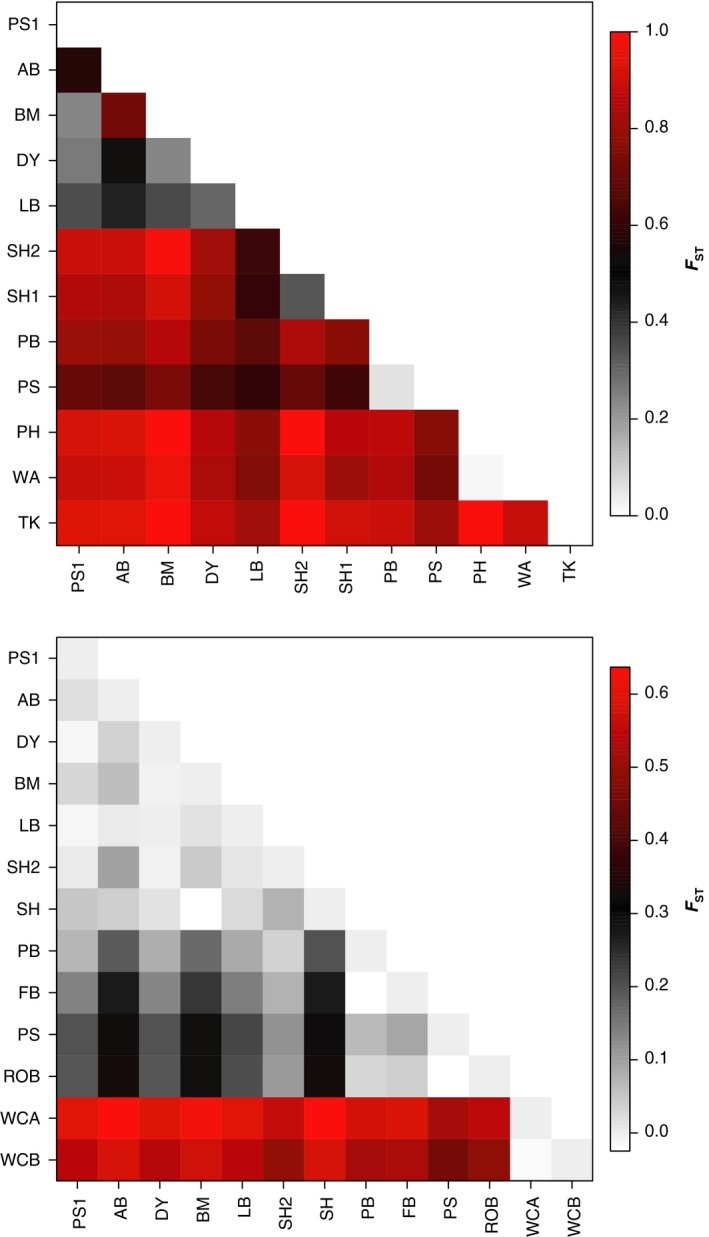
Visual representation of mtDNA pairwise ϕ_ST_ values for *P. exigua* (top panel) and *M. calcar* (bottom panel)

*Parvulastra exigua* displayed genetic structure across all loci and levels of hierarchical sampling. Pairwise values of ϕ_ST_ ranged from 0.018 to 1.00, with 64 of 66 comparisons significant at the *p* < .05 level for mtDNA loci (Figure 5; Table S1). $G_{\mathrm{ST}}^{\prime \prime }$ values ranged from 0.084 to 0.982 for microsatellite loci with all 50 comparisons significant at the *p* < .001 level (Table S2). For nDNA loci, $G_{\mathrm{ST}}^{\prime \prime }$ values ranged from −0.069 to 0.990 with the majority of comparisons significant at the *p* < .05 level (Table S3). AMOVA for all three sets of loci confirm that the majority of the population structure in *P. exigua* is among populations within regions (78.36% mtDNA, 68.80% microsatellite, 71.6% nDNA; Table 2). Grouping populations by geographical region explains less variance than individual populations did, even though Bayesian clustering analyses largely confirmed grouping populations by geography.

**Table 2:** Summary of AMOVAs for both *Parvulastra exigua* and *Meridiastra calcar*

	*P. exigua*	% Variation	*p*‐Value	*M. calcar*	% Variation	*p*‐Value
mtDNA						
By Region	Among Groups	67.21	.001	Among Groups	51.69	.005
	Within Groups	13.21	.001	Within Groups	0.28	.105
	ϕ_CT_	0.6721	.002	ϕ_CT_	0.5197	.005
By Population	Among Populations	78.36	.001	Among Populations	42.12	.001
	Within Populations	21.64		Within Populations	57.88	
	ϕ_ST_	0.7836	.001	ϕ_ST_	0.4212	.001
Microsats						
By Region	Among Groups	23.6	.016	Among Groups	5.2	.025
	Within Individual	23.8		Within Individual	64.2	
	Within Groups	52.7	.001	Within Groups	30.6	.001
	$F_{\mathrm{CT}}^{\prime }$	0.3340	.016	$F_{\mathrm{CT}}^{\prime }$	0.1220	.025
By Population	Among Populations	68.9	.001	Among Populations	20	.001
	Within Individual	24.7		Within Individual	65.6	
	Within Populations	6.3	.001	Within Populations	14.4	.001
	$F_{\mathrm{ST}}^{\prime }$	0.7790	.001	$F_{\mathrm{ST}}^{\prime }$	0.3820	.001
nDNA						
By Region	Among Groups	71.6	.001	Among Groups	0.8	.001
	Within Individual	17.4		Within Individual	62.2	
	Within Groups	11.0	.001	Within Groups	37.0	.001
	$F_{\mathrm{CT}}^{\prime }$	0.8470	.001	$F_{\mathrm{CT}}^{\prime }$	0.1990	.001
By Population	Among Populations	75.7	.001	Among Populations	1.1	.001
	Within Individual	5.1		Within Individual	62.4	
	Within Populations	5.1	.001	Within Populations	36.6	.001
	$F_{\mathrm{ST}}^{\prime }$	0.8590	.001	$F_{\mathrm{ST}}^{\prime }$	0.2490	.001

In contrast, *M. calcar* has low to moderate levels of pairwise population structure, with mtDNA ϕ_ST_ = 0.000–0.637 (Table S4), microsatellite $G_{\mathrm{ST}}^{\prime \prime }=0.017-0.708$ (Table S5), and nDNA $G_{\mathrm{ST}}^{\prime \prime }$ = −0.078 to 0.694 (Table S6). Results from the mtDNA data showed that populations of *M. calcar* exhibited more regional differentiation than *P. exigua* (Figure 5). NSW populations of *M. calcar* are largely similar to each other with only a few significant pairwise comparisons between them. The highest levels of genetic structure were between the SA populations and all other regions. In contrast to the mtDNA data, *M. calcar* has comparatively high pairwise microsatellite $G_{\mathrm{ST}}^{\prime \prime }$ between almost all populations (Table S5) with the nDNA loci indicating strong regional structure but low levels of within‐region structure (Table S6). AMOVA results also show a distinct regional pattern in genetic structure with the majority of microsatellite variation in this species among individuals within populations (Table 2). The three groupings defined by Bayesian clustering (NSW; eastern TAS; western TAS + SA) explained more molecular variance in the mtDNA loci, but explained less of the microsatellite variation.

### 3.5 Coalescent analyses of migration rates, population sizes, and divergence times

Estimates of gene flow from IMa2p were consistent with the strong regional population structure in *P. exigua*. The highest estimated migration rate was 1.35 migrants per generation from population NSW to population SH1, with all others below one migrant per generation (Table 3). Estimates of effective population size were also relatively low (88–6,038 individuals) with the exception of the population from NSW which had an estimated population size of about 70,000 (Table 3).

**Table 3:** IMa2 estimates of *Parvulastra exigua* population parameters with the most probable estimate (MPE) and 95% confidence intervals. Population size is in terms number of individuals, and migration rates are in units of number of effective migrants per generation. Only samples that had no missing data across the five nDNA loci and mtDNA locus were included and samples within northern NSW (AB, PS1; labeled as AB), within central NSW (DY, LB, BM; labeled as NSW), within southern NSW (SH1, SH2; labeled as SH), and within SA (WA, TK; labeled as SA) were combined and randomly subsampled into representative populations

	2.50%	MPE	97.50%
Population Size			
AB	20	88	1,054
NSW	12,096	71,475	616,153
PB	24	68	629
SA	857	2,407	6,050
SH	127	699	2,793
PS	1,489	6,038	14,482
Migration Rates			
AB→NSW	0.14080	0.7846	2.7560
NSW→AB	0.00000	0.4198	110.40
AB→SH	0.00000	0.04536	0.6865
SH→AB	0.00000	0.004626	1.7900
NSW→SH	0.00000	1.347	61.100
SH→NSW	0.00000	0.1358	1.1640
PB→SH	0.00235	0.05239	0.2744
SH→PB	0.00000	0.04105	0.3482
PB→PS	0.14990	0.5449	1.3640
PS→PB	0.00000	0.823	3.5400

In *M. calcar*, estimated migration rates and effective population sizes were one to five orders of magnitude higher than in *P. exigua* (Table 4). Migration rates between northern/central NSW and southern NSW were high in both directions (~40 migrants per generation) and similarly high between eastern and western Tasmania (though only in the east to west direction). Large numbers of migrants were also being exchanged from SA to eastern Tasmanian (~60 migrants per generation), but interestingly not nearly as high between SA and western Tasmania (~4 migrants per generation). In general, migration rates were higher into Tasmania than from Tasmania, although many rates have overlapping confidence intervals. Effective population sizes estimates ranged from ~25,000 individuals in NSW to over 2 million individuals in southern NSW, although all had overlapping confidence intervals with upper bounds well above 500,000 individuals (Table 4).

**Table 4:** IMa2 estimates of *Meridiastra calcar* population parameters with the most probable estimate (MPE) and 95% confidence intervals. Population size is in terms of number of individuals, and migration rates are in units of number of effective migrants per generation. Samples from within northern and central NSW (AB, PS1, DY, LB, BM; labeled as NSW), within southern NSW (SH1, SH2; labeled as SH), within eastern TAS (FB, PB; labeled as FBPB), within western TAS (PS, ROB; labeled as PSROB), and within WA (WCB, WCA; labeled as SA) were combined

	2.50%	MPE	97.50%
Population size			
NSW	6,651	25,274	23,93,013
FBPB	25,495	43,231	2,105,027
SH	1,007,620	2,105,027	6,195,364
PSROB	176,284	411,328	775,268
SA	528,418	823,277	1,461,104
Migrants per generation			
NSW→FBPB	0.000	0.4798	241.3
FBPB→NSW	0.000	0.3748	416.4
NSW→SH	22.31	37.180	4439
SH→NSW	0.000	45.350	405.9
NSW→PSROB	0.000	1.4990	381.7
PSROB→NSW	0.000	0.1424	67.08
FBPB→SH	0.000	0.7496	258.6
SH→FBPB	0.000	5.8470	339.6
FBPB→PSROB	6.559	80.900	3,750
PSROB→FBPB	0.000	1.1750	63.56
FBPB→SA	0.000	15.620	797.7
SA→FBPB	0.000	59.570	97.12
SH→WA	1.040	6.8870	28.20
SA→SH	0.000	1.0250	11.62
PSROB→SA	2.945	7.7960	16.32
SA→PSROB	0.000	4.1850	33.74

**Figure 6:**
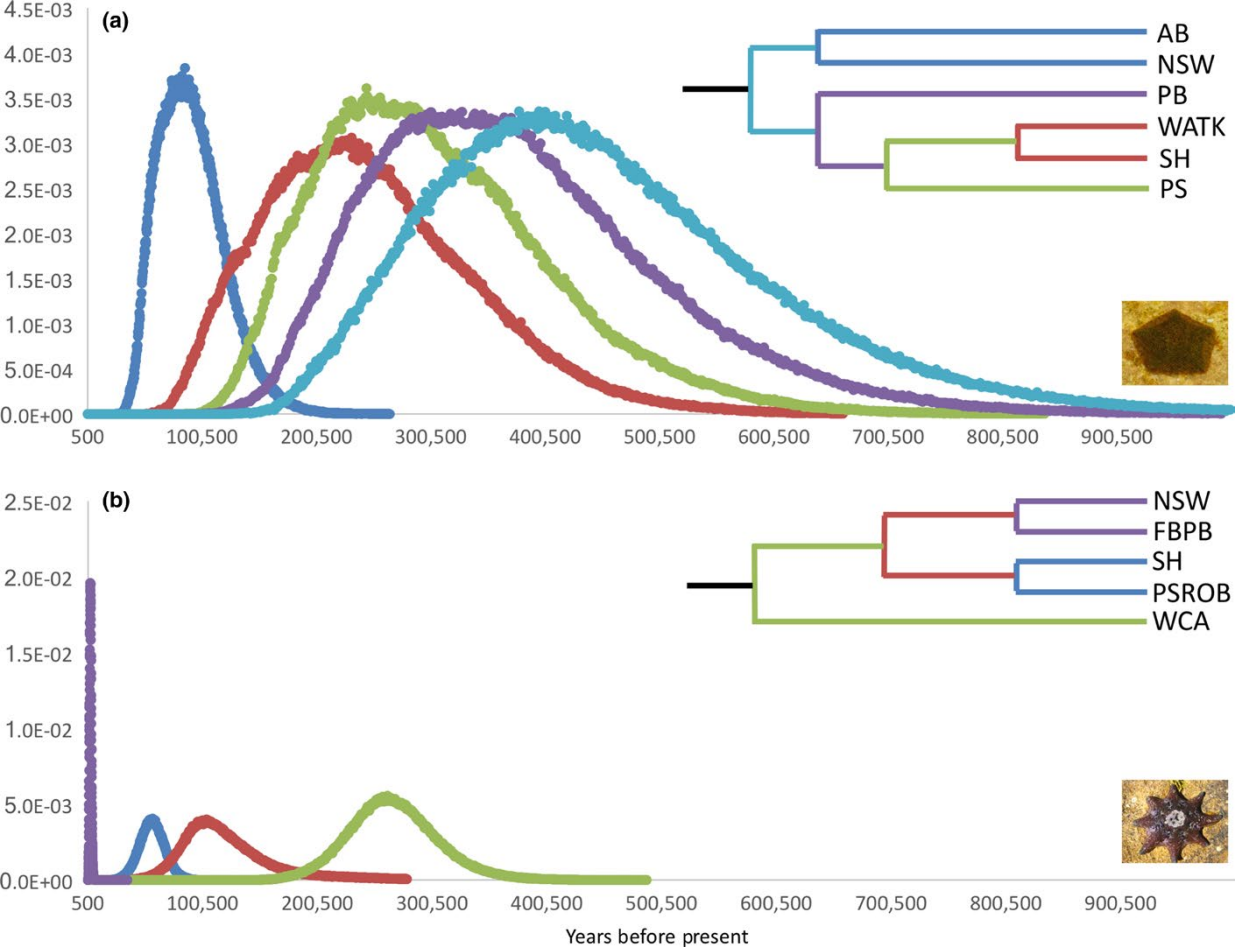
Population divergence time estimates for both species. Population tree used for each coalescent analysis is represented in the upper right corner. (a) *Parvulastra exigua—*Only samples that had no missing data across the five nDNA loci and mtDNA locus were included and samples within northern NSW (AB, PS1; labeled as AB), within central NSW (DY, LB, BM; labeled as NSW), within southern NSW (SH1, SH2; labeled as SH), and within SA (WA, TK; labeled as SA) were combined and randomly subsampled into representative populations. (b) *Meridiastra calcar*—Samples from within northern and central NSW (AB, PS1, DY, LB, BM; labeled as NSW), within southern NSW (SH1, SH2; labeled as SH), within eastern TAS (FB, PB; labeled as FBPB), within western TAS (PS, ROB; labeled as PSROB), and within WA (WCB, WCA; labeled as SA) were combined

Estimates of divergence times between regions differed strikingly between the two species. In *P. exigua*, populations from northern NSW and central NSW diverged only a few thousand years before present (ybp) (2,323; 683–5,962 95% CI); however, that ancestral lineage diverged from all other lineages nearly 20,000 ybp (17,072; 7,488–34,721 95% CI). The other four lineages all diverged approximately 8,000–15,000 ybp (Figure 6a). Notably, southern NSW were inferred as sister populations, separated by only about 8,000 ybp (3,709–20,394). For *M. calcar*, the oldest estimated divergence time was between population SA populations and all other populations nearly 275,000 ybp (272,388; 192,931–375,185 95% CI). The remaining four lineages showed a divergence pattern in contrast to geography (Figure 6b). Southern NSW and the western Tasmania sites diverged from each other about 50,000 ybp (51,665; 29,743–73,905 95% CI), and northern/central NSW and eastern Tasman sites diverged very recently (1,245; 542–2,075 95% CI). The two ancestral populations of the NSW and Tasmanian population pairs diverged nearly 75,000 ybp (74,770; 45,798–142,956 95% CI).

## 4 Discussion

With extensive sympatric ranges that span most of southern and eastern Australia, *M. calcar* and *P. exigua* provide a robust comparative system to study the population genetic effects of their striking life‐history differences. The larger of the two species, *M. calcar*, is a gonochoric, broadcast spawner with a relatively short (10–20 days) nonfeeding pelagic larval stage. In contrast, *P. exigua* has protandry‐like hermaphroditism and benthic larvae, presumably with more limited potential for dispersal. Thus, all else being equal, these differences in reproductive and developmental modes make clear predictions about the degree of gene flow and scale of population genetic structure expected in comparisons of these two species, but results of such comparisons have been somewhat mixed to date (reviewed by Liggins et al., 2016; Mercier et al., 2013). Here, we use the most extensive set of loci for two cosampled marine species and show that our results support the expectation that benthic larval development strongly affects genetic and demographic characteristics of these two intertidal sea star species. The benthic developer, *P. exigua*, showed lower levels of genetic diversity across all markers, more inferred genetic clusters, and higher overall and pairwise population genetic structure compared to the planktonic disperser, *M. calcar*. Additionally, coalescent analyses confirmed extremely low levels of gene flow among all populations of *P. exigua* and between some regional populations of *M. calcar*.

### 4.1 The effects of larval dispersal on population connectivity

Results from this study, and several others on these two species (Ayre et al., 2009; Barbosa et al., 2013; Colgan, Byrne, Rickard, & Castro, 2004; Hunt, 1993; Sherman et al., 2008), have consistently demonstrated that life‐history characteristics predict levels of genetic structure. We showed on several different hierarchical levels and across three different types of genetic markers that the benthic larval development of *P. exigua* leads to higher levels of genetic structure and consistently lower levels of genetic diversity (presumably due to smaller effective population size) when compared to *M. calcar*, a sympatric species with planktonic development. AMOVA and pairwise comparisons of all three marker types show consistently that the majority of genetic variation in *P. exigua* is partitioned among populations. This is not surprising, considering that populations of *P. exigua* can be strongly structured (*F*_ST_ ~ 0.6) on spatial scales of tide pools only meters apart (Barbosa et al., 2013).

*Meridiastra calcar* also displayed relatively high levels of overall and regional genetic structure, especially compared to other asterinid species with planktonic development (Keever et al., 2009; Puritz & Toonen, 2011), but with little genetic differentiation among sites within regions, especially within NSW. Coalescent estimates of within‐region migration line up with recent models of nearshore offshore oceanographic currents (Teske, Sandoval‐Castillo, van Sebille, Waters, & Beheregaray, 2015, 2016), such as the highly unbalanced gene flow seen from eastern to western Tasmania and the high rates of exchange between southern and central NSW. Across‐region estimates of gene flow were generally much lower and corresponded better with offshore current patterns, suggesting that *M. calcar* larvae do, at times, leave the continental shelf. One of the four genetic clusters in both analyses shared individuals from NSW, TAS, and SA, and results from Bayesian clustering indicate that some distant populations show evidence of either recent gene flow or shared ancestry. We suggest three possible explanations for this pattern: (1) SA, based on phylogenetic networks and coalescence population size estimates, may be one of the older, larger, and more ancestral populations of *M. calcar* and this genetic cluster may include many shared ancestral polymorphisms; (2) the two dominant surface currents of southeastern Australia (Figure 1) could promote gene flow into TAS from NSW and WA; (3) sample sizes, both in terms of both number of individuals per locality and number of loci, could not fully resolve the genetic clustering of this highly polymorphic species.

Even with varying levels of genetic structure, *M. calcar* has unusually high levels of genetic diversity, especially for nuclear loci. This nDNA and microsatellite diversity, consistent with previous allozyme analyses (Hunt, 1993; Sherman et al., 2008), eclipses even the high levels of nDNA and microsatellite diversity found in *Patiria miniata*, an asterinid with a long‐lived feeding planktonic larval stage (Keever et al., 2009), and is substantially higher than the diversity levels found in another asterinid species with planktonic lecithotrophic larval development, *Cryptasterina pentagona* (Puritz et al., 2012). In short, our results confirm that life‐history characteristics, especially whether larval development is benthic or pelagic, and their relationship to genetic structure and diversity, are consistent with other previous comparative studies (Barbosa et al., 2013; Dawson et al., 2002; Duffy, 1993; Haney et al., 2008; Hellberg, 1996; Hoskin, 1997; Hunt, 1993; Lee & Boulding, 2009; Riginos & Victor, 2001; Selkoe & Toonen, 2011; Sherman et al., 2008).

The contrasting patterns of phylogeography inferred for these two species provide insight into their recent history and population biology, with the benthic developer showing shallower divergence times between populations than the more broadly dispersing planktonic developer. These results suggest an interesting trade‐off for these life‐history syndromes; despite lower rates of gene flow, the benthic hermaphrodite is actually a better colonizer, and that local extinctions are more easily replaced by new colonists on shorter timescales (Johannesson, 1988). In the planktonic disperser, the populations are less likely to go extinct (both because of larger population size and more recruitment) but when they do go extinct, it takes longer for new colonists to arrive and persist as a population. Further, the mutational distance between low frequency haplotypes in SA for the planktonic disperser indicates that it may have once been a larger population that has recently declined, or that it was once subdivided and is now admixed (Avise, 2004).

### 4.2 The effects of larval dispersal on phylogeography

The spatial phylogeographic patterns and regional genetic structuring of *P. exigua* and *M. calcar* are very similar, despite their dramatically different life histories. However, coalescent analyses estimated substantially different population divergence times for the two species. Linking spatial genetic patterns with population divergence times provides a clearer view of the relationship between life‐history characteristics and phylogeography. Climate and sea‐level changes of the late Pleistocene were particularly powerful influences on the marine fauna of southern Australia (Ayre et al., 2009; Dawson, 2005). When sea levels fell to lower than 50 m below contemporary levels, a land bridge formed between Victoria and Tasmania in the present day area of Bass Strait. The emergence and subsequent submergence of the Tasmanian land bridge would have cut off circulation and gene flow between the Southern Ocean and the Tasman Sea, providing opportunities for reproductive isolation in refugia (Sinclair et al., 2016), differentiation via diversifying selection, and lineage extinctions (Teske, Sandoval‐Castillo, Waters, & Beheregaray, 2016). Several other species show strong genetic breaks or phylogenetic structure at this point (Ayre et al., 2009; Dawson, 2005; Sherman et al., 2008; Waters, 2008; Waters & Roy, 2003; York, Blacket, & Appleton, 2008). Our phylogeographic analyses indicate substantially different recent histories for these two species. *P. exigua* fits a metapopulation scenario of extirpation of many populations during the last glacial maximum with subsequent recolonization of available habitats, whereas populations of *M. calcar* apparently persisted during the last glaciation, with subsequent introgression of alleles between diverged populations after the reopening of seaways.

We estimated the longest divergence time between populations of *P. exigua* at just over 17,000 ybp (7,488–34,721 95% CI), when the ancestral lineage of northern and central NSW diverged from all other populations. The most probable estimate (MPE) corresponds with peaks in relative glacial periods (Billups, 2004). The other sampled populations of *P. exigua* had much shallower divergence times (MPE ~ 2,000–13,000 ybp), well after the end of the last glaciation and reestablishment of contemporary sea surface temperatures and sea levels around 11,000 ybp (Bostock, Opdyke, Gagan, Kiss, & Fifield, 2006). Interestingly, these divergence estimates (with the exception of northern and central NSW) largely precede the reestablishment of the East Australian Current around 5,000 ybp (Bostock et al., 2006), but the confidence intervals of our estimate include this period as well. We hypothesize that during the last glacial maximum, most populations of *P. exigua* in southern Australia were extirpated leaving refugia in northern NSW and either the eastern coast of Tasmania or the southern coast of Victoria. Following the resubmergence of the Tasmanian land bridge, populations in southern NSW, SA, and TAS were then colonized by sporadic successful dispersal events, through rafting or sporadic displacement of the benthic larvae (Bryan et al., 2012; Byrne, 1995; Hart, Byrne, & Smith, 1997; Johannesson, 1988; Thiel & Gutow, 2005; Waters & Roy, 2004) from the southern refugium prior to the reestablishment of the Eastern Australian Current.

For *M. calcar*, the longest divergence time was roughly 275,000 ybp (272,388; 192,931–375,185 95% CI) between SA populations and populations from NSW and TAS. This timing falls between two of the greater glacial peaks during the Pleistocene (~200,000 ybp and ~600,000 ybp; Billups, 2004). Populations of *M. calcar* would have likely started diverging during the later and more extreme glacial maxima, but extant haplotypes may coalesce at a slightly earlier date or the earlier coalescence date is due to inaccuracies in mutation rate estimates. During the LGM, the Eastern Australian Current shifted much farther north, between 23° and 26°S, and may have limited migration between NSW and Tasmanian populations, promoting divergence of these populations. Interestingly, populations between northern/central NSW and western TAS have a very shallow divergence time (~1,000 ybp), and yet have less than one effective migrant per generation between them. In contrast, northern/central NSW and southern NSW are not even inferred to be sister populations, diverging over 75,000 ybp, but have effective migrant numbers well over 35 effective migrants per generation in both directions. We hypothesize that SA populations of *M. calcar* were isolated from NSW and TAS during the majority of the Pleistocene and that populations in NSW and TAS were restricted to two ancestral refugia during the LGM; present day populations have ancestry from one of these two ancestral lineages, but are being homogenized by moderate levels of gene flow within NSW and TAS, with low gene flow between regions mediated by the contemporary sea surface currents.

Our results with multiple genetic markers augment the recent study using mtDNA COI that showed that life‐history characteristics failed to predict the effect of Bass Strait, a known phylogeographic barrier affecting the population structure of several intertidal species (Ayre et al., 2009). In this previous study, COI haplotypes of *M. calcar* were significantly differentiated across the Strait, whereas genetic structure in *P. exigua* was entirely among populations, such that grouping populations into east and west of Bass Strait in a hierarchical AMOVA explained no additional molecular variance over populations alone. Our combined mtDNA, microsatellite, and nDNA results show that virtually all genetic variation is partitioned at the population, and not regional, level in *P. exigua*, clarifying the findings based on COI haplotypes alone. In addition, our coalescent analyses on the combined dataset help to explain previous findings because they show that populations east and west of Bass Strait all share a post‐Pleistocene origin. In that sense, the Tasmanian land bridge was not a barrier for *P. exigua*. This example highlights the value of multiple marker studies incorporating coalescent analyses to further our understanding of population connectivity in marine species. Our findings indicate that phylogeographic patterns in *P. exigua* and *M. calcar* are consistent with differences in their life histories, and this result is consistent with a previous meta‐analysis showing that the divergence between benthic and pelagic development was the primary correlate between life‐history differences and population structure (Weersing & Toonen, 2009).

## 5 Conclusion

This study shows that incorporating multiple marker types provides a more complete understanding of the contrasting local and regional population connectivity of these two sympatric species than single‐marker approaches, and together with explicit modeling of historical processes helps to makes sense of otherwise apparently contrasting results among studies. Additionally, we show that populations of *P. exigua* are each evolving essentially independently, with the majority of genetic diversity limited to the single population scale. Moreover, our phylogeographic results indicate that this species may be particularly sensitive to changes in sea levels, with strong subsequent effects on both patterns of extirpation and recolonization as well as the resultant population structure. Within the current context of global ocean and climate change, these results have implications for conservation of these species, and likely also for other species in the region that lack pelagic larvae.

## Conflict of Interest

The authors declare that they have no conflict of interest.

## Data Accessibility

Raw 454 files, nDNA and microsatellite genotypes, mtDNA alignments, and IMa2 input and output files can be found on Figshare.https://figshare.com/projects/Life_History_Predicts_Past_and_Present_Population_Connectivity_in_Two_Sympatric_Sea_Stars/17007).
