# Supplementary material for: Life‐history predicts past and present population connectivity in two sympatric sea stars
Source: Ecol Evol. 2017 Apr 23;7(11):3916–30. doi: 10.1002/ece3.2938 (PMC5468144; doi:10.1002/ece3.2938)
Supplement: Supplementary file 1 [file ECE3-7-3916-s001.docx]

**Supplementary Materials**

Supplemental Figures:

Figure S1. Plot of estimated likelihoods against K for *Meridiastra calcar* STRUCTURE runs.

Table S1. Pairwise ***ϕ_ST_*** values for *Parvulastra exigua* from mtDNA loci. Bold values have P-values < 0.05.

|  | PS1 | AB | BM | DY | LB | SH2 | SH1 | PB | PS | PH | WA |
| --- | --- | --- | --- | --- | --- | --- | --- | --- | --- | --- | --- |
| PS1 |  |  |  |  |  |  |  |  |  |  |  |
| AB | **0.577** |  |  |  |  |  |  |  |  |  |  |
| BM | **0.237** | **0.719** |  |  |  |  |  |  |  |  |  |
| DY | **0.260** | **0.479** | **0.240** |  |  |  |  |  |  |  |  |
| LB | **0.345** | **0.433** | **0.353** | **0.302** |  |  |  |  |  |  |  |
| SH2 | **0.891** | **0.898** | **0.990** | **0.819** | **0.621** |  |  |  |  |  |  |
| SH1 | **0.845** | **0.838** | **0.912** | **0.787** | **0.609** | **0.328** |  |  |  |  |  |
| PB | **0.797** | **0.790** | **0.853** | **0.736** | **0.686** | **0.839** | **0.767** |  |  |  |  |
| PS | **0.700** | **0.687** | **0.739** | **0.633** | **0.594** | **0.698** | **0.631** | 0.062 |  |  |  |
| PH | **0.914** | **0.928** | **0.992** | **0.856** | **0.771** | **1.000** | **0.860** | **0.873** | **0.759** |  |  |
| WA | **0.889** | **0.897** | **0.958** | **0.834** | **0.751** | **0.914** | **0.806** | **0.845** | **0.734** | 0.018 |  |
| TK | **0.930** | **0.938** | **0.994** | **0.881** | **0.815** | **1.000** | **0.906** | **0.897** | **0.805** | **1.000** | **0.886** |

Table S2. Pairwise *G’’_ST_* values for *Parvulastra exigua* from microsatellite loci. All comparisons have P-values < 0.0001.

|  | PS1 | DY | BM | LB | SH2 | SH1 | PB | PS | PH |
| --- | --- | --- | --- | --- | --- | --- | --- | --- | --- |
| DY | 0.557 |  |  |  |  |  |  |  |  |
| BM | 0.847 | 0.739 |  |  |  |  |  |  |  |
| LB | 0.084 | 0.376 | 0.769 |  |  |  |  |  |  |
| SH2 | 0.607 | 0.142 | 0.751 | 0.475 |  |  |  |  |  |
| SH1 | 0.462 | 0.193 | 0.695 | 0.316 | 0.138 |  |  |  |  |
| PB | 0.913 | 0.742 | 0.811 | 0.853 | 0.723 | 0.701 |  |  |  |
| PS | 0.913 | 0.804 | 0.771 | 0.877 | 0.810 | 0.802 | 0.846 |  |  |
| PH | 0.985 | 0.845 | 0.955 | 0.927 | 0.851 | 0.859 | 0.955 | 0.960 |  |
| TK | 0.982 | 0.918 | 0.866 | 0.953 | 0.923 | 0.916 | 0.945 | 0.922 | 0.888 |

Table S3. Pairwise *G’’_ST_* values for *Parvulastra exigua* from nDNA loci. Bold indicates P-values < 0.05 and bold plus italics indicates P-values < 0.0001.

|  | | PS1 | | | AB | | | DY | | BM | | | LB | | | SH2 | | | SH1 | PB | PS | PH | TK |
| --- | --- | --- | --- | --- | --- | --- | --- | --- | --- | --- | --- | --- | --- | --- | --- | --- | --- | --- | --- | --- | --- | --- | --- |
| AB | | -0.069 | | |  | | |  | |  | | |  | | |  | | |  |  |  |  |  |
| DY | | ***0.650*** | | | ***0.635*** | | |  | |  | | |  | | |  | | |  |  |  |  |  |
| BM | | **0.113** | | | 0.044 | | | ***0.438*** | |  | | |  | | |  | | |  |  |  |  |  |
| LB | | **0.163** | | | 0.124 | | | ***0.283*** | | 0.038 | | |  | | |  | | |  |  |  |  |  |
| SH2 | | ***0.296*** | | | **0.227** | | | ***0.520*** | | 0.046 | | | ***0.195*** | | |  | | |  |  |  |  |  |
| SH1 | | ***0.681*** | | | ***0.633*** | | | ***0.754*** | | ***0.359*** | | | ***0.537*** | | | ***0.143*** | | |  |  |  |  |  |
| PB | | ***0.776*** | | | ***0.763*** | | | ***0.848*** | | ***0.661*** | | | ***0.736*** | | | ***0.625*** | | | ***0.715*** |  |  |  |  |
| PS | | ***0.819*** | | | ***0.805*** | | | ***0.869*** | | ***0.692*** | | | ***0.766*** | | | ***0.646*** | | | ***0.739*** | ***0.601*** |  |  |  |
| PH | | ***0.981*** | | | ***0.981*** | | | ***0.972*** | | ***0.965*** | | | ***0.966*** | | | ***0.964*** | | | ***0.984*** | ***0.956*** | ***0.973*** |  |  |
| TK | | ***0.990*** | | | ***0.989*** | | | ***0.981*** | | ***0.974*** | | | ***0.975*** | | | ***0.973*** | | | ***0.992*** | ***0.969*** | ***0.983*** | **0.162** |  |
| WA | | ***0.983*** | | | ***0.983*** | | | ***0.974*** | | ***0.967*** | | | ***0.967*** | | | ***0.965*** | | | ***0.986*** | ***0.960*** | ***0.976*** | 0.052 | 0.025 |
|  |  | |  |  | |  |  | |  | |  |  | |  |  | |  |  |  |  |  |  |  |
|  |  | |  |  | |  |  | |  | |  |  | |  |  | |  |  |  |  |  |  |  |

Table S4. Pairwise ***ϕ_ST_*** values for *Meridiastra calcar* from mtDNA loci. Bold values have P-values < 0.05.

|  | PS1 | AB | DY | BM | LB | SH2 | SH | PB | FB | PS | ROB | WCA |
| --- | --- | --- | --- | --- | --- | --- | --- | --- | --- | --- | --- | --- |
| PS1 |  |  |  |  |  |  |  |  |  |  |  |  |
| AB | 0.017 |  |  |  |  |  |  |  |  |  |  |  |
| DY | 0.000 | **0.035** |  |  |  |  |  |  |  |  |  |  |
| BM | 0.029 | **0.061** | 0.000 |  |  |  |  |  |  |  |  |  |
| LB | 0.000 | 0.002 | 0.000 | 0.015 |  |  |  |  |  |  |  |  |
| SH2 | 0.003 | **0.098** | 0.000 | 0.043 | 0.009 |  |  |  |  |  |  |  |
| SH | **0.050** | 0.038 | **0.013** | 0.000 | 0.024 | **0.077** |  |  |  |  |  |  |
| PB | **0.073** | **0.187** | **0.083** | **0.169** | **0.086** | 0.037 | **0.201** |  |  |  |  |  |
| FB | **0.138** | **0.270** | **0.132** | **0.238** | **0.142** | **0.078** | **0.272** | 0.000 |  |  |  |  |
| PS | **0.203** | **0.322** | **0.202** | **0.291** | **0.216** | **0.117** | **0.320** | 0.066 | **0.092** |  |  |  |
| ROB | **0.196** | **0.328** | **0.192** | **0.294** | **0.204** | **0.106** | **0.327** | 0.030 | 0.042 | 0.000 |  |  |
| WCA | **0.598** | **0.637** | **0.593** | **0.626** | **0.599** | **0.554** | **0.632** | **0.579** | **0.588** | **0.519** | **0.550** |  |
| WCB | **0.544** | **0.585** | **0.542** | **0.572** | **0.548** | **0.497** | **0.580** | **0.520** | **0.526** | **0.457** | **0.489** | 0.000 |

Table S5. Pairwise *G’’_ST_* values for *Meridiastra calcar* from microsatellite loci. All comparisons have P-values < 0.001 except for DY-SH2 (p=0.074) and WCA-WCB (p=0.049).

|  | PS1 | AB | DY | BM | LB | SH2 | PB | FB | PS | WCA |
| --- | --- | --- | --- | --- | --- | --- | --- | --- | --- | --- |
| AB | 0.232 |  |  |  |  |  |  |  |  |  |
| DY | 0.457 | 0.344 |  |  |  |  |  |  |  |  |
| BM | 0.670 | 0.490 | 0.324 |  |  |  |  |  |  |  |
| LB | 0.448 | 0.360 | 0.305 | 0.607 |  |  |  |  |  |  |
| SH2 | 0.440 | 0.361 | *0.017* | 0.391 | 0.277 |  |  |  |  |  |
| PB | 0.480 | 0.227 | 0.191 | 0.238 | 0.397 | 0.265 |  |  |  |  |
| FB | 0.333 | 0.134 | 0.275 | 0.447 | 0.308 | 0.279 | 0.203 |  |  |  |
| PS | 0.595 | 0.386 | 0.187 | 0.110 | 0.550 | 0.292 | 0.125 | 0.384 |  |  |
| WCA | 0.708 | 0.529 | 0.313 | 0.216 | 0.662 | 0.418 | 0.267 | 0.518 | 0.074 |  |
| WCB | 0.687 | 0.499 | 0.280 | 0.218 | 0.637 | 0.392 | 0.214 | 0.487 | 0.048 | *0.022* |

Table S6. Pairwise *G’’_ST_* values for *Meridiastra calcar* from nDNA loci. *G’’_ST_* values on the lower diagonal and P-values on the upper diagonal.

|  | PS1 | AB | DY | BM | LB | SH2 | SH1 | PB | FB | PS | ROB | WCA | WCB |
| --- | --- | --- | --- | --- | --- | --- | --- | --- | --- | --- | --- | --- | --- |
| PS1 | -- | 0.484 | 0.065 | 0.142 | 0.066 | 0.386 | 0.085 | 0.006 | 0.027 | 0.000 | 0.010 | 0.002 | 0.002 |
| AB | -0.011 | -- | 0.457 | 0.482 | 0.176 | 0.103 | 0.112 | 0.007 | 0.052 | 0.001 | 0.001 | 0.002 | 0.003 |
| DY | 0.138 | 0.001 | -- | 0.017 | 0.379 | 0.003 | 0.002 | 0.002 | 0.001 | 0.000 | 0.000 | 0.000 | 0.009 |
| BM | 0.080 | -0.008 | 0.225 | -- | 0.003 | 0.300 | 0.016 | 0.000 | 0.011 | 0.000 | 0.000 | 0.000 | 0.016 |
| LB | 0.154 | 0.112 | 0.030 | 0.287 | -- | 0.003 | 0.067 | 0.000 | 0.001 | 0.000 | 0.000 | 0.000 | 0.003 |
| SH2 | 0.010 | 0.114 | 0.281 | 0.030 | 0.321 | -- | 0.266 | 0.000 | 0.022 | 0.000 | 0.000 | 0.001 | 0.000 |
| SH1 | 0.095 | 0.110 | 0.285 | 0.175 | 0.133 | 0.043 | -- | 0.000 | 0.003 | 0.000 | 0.000 | 0.000 | 0.000 |
| PB | 0.277 | 0.340 | 0.406 | 0.437 | 0.501 | 0.402 | 0.547 | -- | 0.003 | 0.786 | 0.776 | 0.123 | 0.053 |
| FB | 0.194 | 0.198 | 0.408 | 0.241 | 0.467 | 0.195 | 0.287 | 0.347 | -- | 0.001 | 0.000 | 0.025 | 0.005 |
| PS01 | 0.541 | 0.459 | 0.593 | 0.542 | 0.694 | 0.470 | 0.591 | -0.078 | 0.418 | -- | 0.301 | 0.430 | 0.045 |
| ROB01 | 0.189 | 0.355 | 0.409 | 0.378 | 0.484 | 0.328 | 0.394 | -0.057 | 0.352 | 0.024 | -- | 0.110 | 0.006 |
| WCA01 | 0.330 | 0.351 | 0.407 | 0.356 | 0.432 | 0.284 | 0.360 | 0.111 | 0.220 | 0.000 | 0.093 | -- | 0.065 |
| WB01 | 0.386 | 0.497 | 0.388 | 0.308 | 0.411 | 0.462 | 0.569 | 0.225 | 0.389 | 0.236 | 0.285 | 0.198 | -- |
